# Serum Osteopontin and Procollagen Type 1 N-Terminal Propeptide Concentrations: Links to Liver Function, Muscle Mass, and Bone Mineral Density in MASLD and Hypertension

**DOI:** 10.3390/metabo15070459

**Published:** 2025-07-06

**Authors:** Anna F. Sheptulina, Anastasia Yu. Elkina, Elvira M. Mamutova, Yuriy S. Timofeev, Victoria A. Metelskaya, Oxana M. Drapkina

**Affiliations:** 1 Department of Fundamental and Applied Aspects of Obesity, National Medical Research Center for Therapy and Preventive Medicine, 101990 Moscow, Russia; hromyh.anastasiya@mail.ru (A.Y.E.); emamutova@gnicpm.ru (E.M.M.); timofeev_lab@mail.ru (Y.S.T.); vmetelskaya@gnicpm.ru (V.A.M.); drapkina@bk.ru (O.M.D.); 2 Department of Intermediate Level Therapy, Saratov State Medical University, 410012 Saratov, Russia

**Keywords:** metabolic dysfunction-associated steatotic liver disease (MASLD), hypertension (HTN), bone mineral density (BMD), osteopenia, osteoporosis, bone turnover markers (BTMs), osteopontin (OPN), procollagen type 1 N-terminal propeptide (P1NP)

## Abstract

Background/Objectives: Increasing evidence suggests that metabolic dysfunction-associated steatotic liver disease (MASLD) and hypertension (HTN), a well-established cardiometabolic risk factor, both negatively impact bone metabolism. This study aimed to investigate the associations between bone turnover markers (BTMs)—namely, osteopontin (OPN) and procollagen type 1 N-terminal propeptide (P1NP)—and metabolic health indicators, non-invasive measures of liver disease severity, as well as skeletal muscle mass (SMM), muscle strength, and bone mineral density (BMD) in patients with MASLD and HTN. Methods: We enrolled 117 patients diagnosed with MASLD and HTN and conducted anthropometric measurements, laboratory analyses, abdominal ultrasound, and point shear-wave elastography. Muscle strength was evaluated using grip strength measurements and the Five Times Sit-to-Stand Test (FTSST). SMM and BMD were quantified using dual-energy X-ray absorptiometry (DEXA). Serum OPN and P1NP concentrations were quantified using enzyme-linked immunosorbent assays (ELISAs). Results: Serum OPN concentrations below 2.89 ng/mL were associated with significantly elevated levels of AST (*p* = 0.001), ALT (*p* = 0.006), and GGT (*p* = 0.025), while serum P1NP concentrations above 47.5 pg/mL were associated only with significantly elevated GGT levels (*p* = 0.024). In addition, patients with MASLD and HTN with lower serum OPN levels had higher liver stiffness values (*p* = 0.003). Serum OPN concentrations were inversely associated with the following metabolic health indicators: waist circumference (WC, *p* < 0.001) and epicardial fat thickness (EFT, *p* = 0.001). In addition, they were significantly elevated in patients with MASLD and HTN who had decreased spinal BMD (*p* = 0.017). In turn, serum P1NP levels were reduced in patients with decreased SMM (*p* = 0.023). Conclusions: These findings in patients with MASLD and HTN suggest an association between serum P1NP levels and SMM, and between OPN levels and spinal BMD, indicating a potential interplay among liver function, muscle mass, and bone health. Furthermore, OPN appeared to be strongly associated with overall metabolic health indicators, such as WC and EFT, whereas P1NP exhibited a stronger association with muscle mass.

## 1. Introduction

The increasing global adoption of Western diets, combined with sedentary lifestyles and rising obesity rates, has led to a significant increase in the prevalence of metabolic dysfunction-associated steatotic liver disease (MASLD), which now affects 25–37.8% of the population [1,2]. This rise in MASLD incidence parallels an increased prevalence of associated metabolic comorbidities, including hypertension (HTN) [3], type 2 diabetes mellitus (T2D) [4], obesity, and hyperlipidemia [5,6], collectively contributing to the development of multi-organ metabolic syndrome [7].

Hypertension is frequently observed as a comorbidity in patients with MASLD. Moreover, HTN is a recognized cardiometabolic risk factor, and the presence of at least one such risk factor is required for a diagnosis of MASLD, according to the 2023 diagnostic criteria [8]. A 2022 meta-analysis and systematic review encompassing 11 observational studies conducted in the USA, Asia, and Europe (n = 390,348; 52% male, 48% female) demonstrated that MASLD is associated with a 66% increased risk of developing HTN [9]. Conversely, MASLD is significantly more prevalent among individuals with obesity and HTN (30.9%) compared to those with normal blood pressure (12.7%) [10].

Growing evidence indicates that both MASLD [9,11,12,13] and HTN [14] can negatively impact bone metabolism. Specifically, MASLD is associated with decreased bone mineral density (BMD) and an elevated risk of osteoporosis and osteoporotic fractures [9]. Hepatic osteodystrophy, a metabolic bone disease secondary to chronic liver disease, represents a significant but frequently underrecognized complication [11,12]. The prevalence of osteoporosis in patients with MASLD ranges from 10% to 40%, substantially exceeding that of the general population. Furthermore, the presence of MASLD is associated with a 2.5-fold increased risk of osteoporotic fractures, and lower BMD values correlate with more pronounced hepatic disease activity [9,13,15].

Concerning HTN, a meta-analysis by Z. Ye et al. (2017) [14], encompassing 17 studies and 39,491 patients (13,375 with essential HTN and 26,116 with secondary HTN), indicated that elevated blood pressure is associated with an increased risk of decreased BMD. Moreover, the extent of BMD reduction varied depending on the skeletal site and geographic region [14]. In another meta-analysis, C. Li et al. (2017) [16] found that osteoporotic fractures were 33% more prevalent in over 1.4 million patients with arterial hypertension compared to those without. This association was consistent across populations in Asia and Europe [16].

The pathogenesis of decreased BMD in MASLD, with or without coexisting HTN, is multifactorial, including liver-derived pro-inflammatory cytokines, vitamin D deficiency, and reduced physical activity [17]. Recent research has also focused on osteokines, biologically active molecules synthesized and secreted by bone tissue, specifically osteopontin (OPN) and procollagen type 1 N-terminal propeptide (P1NP). Notably, these markers are implicated not only in bone metabolism but also in the development and progression of MASLD and HTN. Indeed, elevated plasma OPN levels have been reported in patients with MASLD, with a positive correlation between OPN concentration and the stage of liver fibrosis [18,19]. Furthermore, associations between serum OPN concentrations and hepatic failure, portal hypertension, and hepatocellular carcinoma (HCC) have also been reported [18,20,21,22]. Consequently, OPN is being investigated as a potential therapeutic target in chronic liver diseases and HCC.

Increasing evidence underscores the important role of osteopontin (OPN) as a mediator of inflammation and vascular remodeling, particularly in large arteries. For instance, K. Miyoshi et al. demonstrated that plasma OPN concentrations may serve as a useful marker for predicting improvements in aortic stiffness (assessed via the augmentation index) during antihypertensive therapy [23]. In another study, using both OPN knockout and wild-type mice with hypertension induced by a 7-day infusion of angiotensin II, the authors found that aortas from OPN knockout mice were protected from angiotensin II-induced medial hypertrophy and inflammation. This protective effect occurred despite similar increases in systolic blood pressure observed in both wild-type and OPN knockout groups [24].

Regarding P1NP, M. Luger et al. demonstrated a significant association between its serum concentrations and the stage of liver fibrosis in postmenopausal women with T2D [25]. Elevated P1NP levels were associated with a 3.65-fold increased risk of advanced liver fibrosis. In addition, in patients with a mean body mass index (BMI) of 44 kg/m^2^, more advanced stages of liver fibrosis were independently associated with elevated P1NP levels [25]. Separately, Y. Mao et al. demonstrated that the administration of specific antihypertensive drugs—namely, hydrochlorothiazide and benazepril—to spontaneously hypertensive ovariectomized rats led to a significant restoration of P1NP and C-terminal telopeptide of type I collagen (CTX) concentrations, along with improvements in bone microarchitecture [26].

Cumulatively, the aforementioned data suggest that (1) MASLD and HTN are associated with decreased BMD, (2) bone turnover markers, OPN and P1NP, are not only involved in the regulation of bone metabolism but also play a key role in the progression of MASLD and vascular remodeling in HTN, and (3) the coexistence of osteoporosis with MASLD and HTN exacerbates long-term morbidity and negatively affects patients’ quality of life [27]. In contrast, physical exercise, a cornerstone of lifestyle modification for both MASLD and HTN, favorably impacts bone health by increasing BMD. However, caution is advised, as certain physical activities (e.g., running, jumping, specific yoga poses, forward bends, torso twists, tennis, and bowling) may elevate the risk of fractures in individuals with osteopenia or osteoporosis [28]. In clinical practice, two key considerations are paramount in the management of patients with MASLD, including those with co-existing conditions, such as HTN: (1) assessing BMD, particularly in patients with additional risk factors for osteopenia/osteoporosis (e.g., postmenopausal women, individuals with a history of osteoporosis-inducing medication use, and those with sarcopenic obesity); and (2) providing personalized recommendations regarding appropriate and beneficial physical activities and dietary interventions. Therefore, investigating serum levels of osteokines, such as OPN and P1NP, in patients with MASLD and HTN may help clarify their association with decreased BMD. This could facilitate the use of these biomarkers for diagnosing osteopenia or osteoporosis, particularly in settings where dual-energy X-ray absorptiometry (DEXA) is unavailable or contraindicated (e.g., patients weighing over 130 kg, pregnant women) [29].

This study aimed to investigate serum concentrations of OPN and P1NP in patients with MASLD and HTN and examine their associations with metabolic health indicators, non-invasive measures of liver disease severity, as well as SMM, muscle strength, and BMD.

## 2. Materials and Methods

### 2.1. Patients

All consecutive, unselected patients with both MASLD and HTN who presented to the outpatient department of the National Medical Research Center for Therapy and Preventive Medicine, Ministry of Healthcare of the Russian Federation, between January 2024 and January 2025 were enrolled in this single-center, cross-sectional study. Patients were included if they met the inclusion criteria and did not meet any of the exclusion criteria. The study protocol was approved by the local Ethics Committee (protocol No. 01-03/20, 23 January 2020). Written informed consent was obtained from all participants prior to enrollment.

• Inclusion criteria:

○ Age 20–70 years.
○ Sonographic evidence of liver steatosis.
○ Absence of other causes of liver steatosis.
○ Established diagnosis of HTN.
○ Signed informed consent.

Exclusion criteria: concomitant liver disease; alcohol abuse (defined as a score ≥ 8 on the Russian version of the Alcohol Use Disorders Identification Test [RUS-AUDIT]); mental disorders; acute infectious diseases; exacerbation of chronic non-communicable diseases (within four weeks prior to enrollment); active oncologic disease without curative treatment; uncontrolled HTN; inflammatory bowel disease; type 1 diabetes mellitus; hypoparathyroidism; chronic kidney disease requiring dialysis; pregnancy or breastfeeding; lower limb fractures within 6 months before this study with ongoing functional limitations; any clinically significant disorders or diseases that impair mobility or self-care; and the absence of signed consent.

All study procedures, including patient interviews, physical examinations, anthropometric measurements (waist circumference (WC) and hip circumference (HC), height, weight), laboratory tests (including complete blood count, biochemical panel, and assessment of serum osteokine concentrations), abdominal ultrasound, and point shear-wave elastography of the liver (using the Philips Affiniti 70 device; Philips, Amsterdam, the Netherlands) were performed during a single visit to the study center.

### 2.2. Diagnosis of Metabolic Dysfunction-Associated Steatotic Liver Disease and Hypertension

The diagnosis of MASLD was based on the 2023 expert consensus statement, requiring the presence of the following three key criteria: (1) sonographic evidence of hepatic steatosis (increased echogenicity of the liver compared to the renal cortex, decreased visibility of vessels within the liver parenchyma, and/or attenuation of the ultrasound signal at the liver periphery); (2) at least one cardiometabolic risk factor (detailed elsewhere [8]); and (3) exclusion of other etiologies of steatotic liver disease. This latter criterion primarily applied to patients with serum ALT activity exceeding 33 U/L (males) or 25 U/L (females), who underwent further testing to rule out other causes of hepatic steatosis (i.e., markers of hepatitis B and C viruses) [30]. Furthermore, all patients completed the RUS-AUDIT questionnaire and were excluded from the study if their score was ≥8.

To further assess the likelihood of hepatic steatosis, the Fatty Liver Index (FLI) was calculated [31]. An FLI value less than 30 (negative likelihood ratio = 0.2) was considered effective in ruling out hepatic steatosis, whereas an FLI value of ≥60 (positive likelihood ratio = 4.3) was considered indicative of hepatic steatosis [31].

The degree of liver fibrosis was assessed using point shear-wave elastography, adhering to established reliability criteria for liver stiffness assessment [32,33].

Hypertension was diagnosed based on patient history and available medical records and classified as: grade 1 HTN (systolic blood pressure [SBP] 140–159 mmHg and/or diastolic blood pressure [DBP] 90–99 mmHg); grade 2 HTN (SBP 160–179 mmHg and/or DBP 100–109 mmHg); and grade 3 HTN (SBP ≥ 180 mmHg and/or DBP ≥ 110 mmHg) [34].

### 2.3. Anthropometric Measurements

Body weight was measured using electronic floor medical scales (VMEN-200-50/100-I-ST-A, TVES LLC, Tambov Oblast, Tambov, Russia) placed on a flat, smooth surface. Height was measured using a stadiometer (R-St-MSK, MSK-234; Medstalkonstruktsiya LLC, Ufa, Russia) following standard procedures. Body mass index (BMI) was calculated as body weight (kg) divided by height squared (m^2^).

Waist circumference (WC) and hip circumference (HC) were measured in standing patients, ensuring the stomach was relaxed, arms were loosely lowered, and heels were together. WC was measured with a tape measure at the narrowest point of the abdomen (i.e., the natural waist) at the end of a normal exhalation, with the tape held snug against the clothing but without compressing the skin. HC was measured by placing the tape measure around the hips at the point of maximum protrusion of the buttocks.

### 2.4. Skeletal Muscle Mass and Bone Mineral Density Assessment

Skeletal muscle mass and BMD were assessed using dual-energy X-ray absorptiometry (DEXA). The following DEXA parameters were recorded: skeletal muscle mass (SMM); appendicular skeletal muscle mass (ALM, i.e., SMM from both legs and arms); and lumbar spine and femoral neck BMD. To assess for reduced SMM, the appendicular lean mass-to-body weight ratio (ALM/W) was calculated from DEXA results, representing the sum of lean muscle mass in the upper and lower limbs, adjusted for patient weight. Reduced SMM was defined as ALM/W values of <28.27% for males and <23.47% for females [35]. Osteopenia was diagnosed according to World Health Organization criteria (T-scores ranging from −1.0 to −2.5), and osteoporosis was diagnosed when T-scores were ≤−2.5 [36].

### 2.5. Muscle Strength Assessment

Arm and leg muscle strength were assessed by measuring grip strength (GS) and administering the Five Times Sit-to-Stand Test (FTSST) [37,38]. GS of the dominant hand was measured using a hand-held mechanical dynamometer (DK-50, NTMIZ CJSC, Nizhny Tagil, Russia). Patients were instructed to squeeze the dynamometer as forcefully as possible on three separate occasions, with a 60 s rest between each attempt. Decreased GS was defined as ≤30 kg for males with a BMI of 26.1 to 28 kg/m^2^, ≤32 kg for males with a BMI > 28 kg/m^2^, ≤18 kg for females with a BMI of 26.1 to 29 kg/m^2^, and ≤21 kg for females with a BMI > 29 kg/m^2^ [37].

All patients also performed the FTSST, following the methodology described previously [39,40]. Prolonged completion of the FTSST (i.e., >15 s) was indicative of decreased muscle strength [39].

### 2.6. Quantification of Serum Osteopontin and Procollagen Type 1 N-Terminal Propeptide

Serum OPN and P1NP levels were quantified using direct solid-phase enzyme-linked immunosorbent assays (ELISAs) with standardized test systems.

Serum OPN levels were measured using the ELISA Kit for Osteopontin (Cloud-Clone Corp., Wuhan, China/ Katy, TX, USA), which had a calibration range of 0.625–40.0 ng/mL and an analytical sensitivity of 0.237 ng/mL. P1NP levels were measured using the ELISA Kit for Procollagen Type 1 N-Terminal Propeptide (P1NP) (Cloud-Clone Corp., Wuhan, China/ Katy, TX, USA), following the manufacturer’s recommended dilutions with phosphate-buffered saline (PBS, 0.01 mmol/L, pH 7.0–7.2), and an analytical sensitivity of 34 pg/mL.

### 2.7. Statistical Analysis

Data distribution was assessed using the Kolmogorov–Smirnov test. Nonparametric tests were employed because the data were not normally distributed. Categorical variables are presented as n (%) and continuous variables as medians with interquartile ranges (IQRs; 25th, 75th percentiles). Between-group differences were assessed using the Mann–Whitney *U* test, Kruskal–Wallis test, or Spearman’s rank correlation coefficient, as appropriate. Correlation strength was categorized according to the Chaddock scale: negligible (0.1 ≤ *ρ* < 0.3), weak (0.3 ≤ *ρ* < 0.5), moderate (0.5 ≤ *ρ* < 0.7), strong (0.7 ≤ *ρ* < 0.9), and very strong (0.9 ≤ *ρ* < 1.0) [41]. Pearson’s chi-squared test (χ^2^) was used for categorical variables. To explore the potential associations between serum concentrations of OPN and P1NP and metabolic health indicators, non-invasive measures of liver disease severity, as well as muscle strength, SMM, and BMD, we stratified patients into groups based on “lower” and “higher” concentrations of these markers. As no established clinical cutoffs exist for these markers, we applied an ad hoc approach by visually inspecting the distribution of values using histograms and kernel density plots. The approximate peak of each distribution was used as a reference point to define the cutoff for stratification. It should be noted that this approach does not rely on the statistical mode in the strict sense, but rather on the most prominent density peak, and is intended solely for exploratory purposes. Statistical significance was set at *p* < 0.05 for all analyses. Statistical analyses were performed using IBM SPSS Statistics, version 27.0 (IBM Corp., Armonk, NY, USA).

## 3. Results

### 3.1. Patient Characteristics

Between January 2024 and January 2025, 138 patients diagnosed with MASLD and HTN who met this study’s inclusion criteria were evaluated at the Department of Clinical Diagnostics, National Medical Research Center for Therapy and Preventive Medicine.

**Figure 1:**
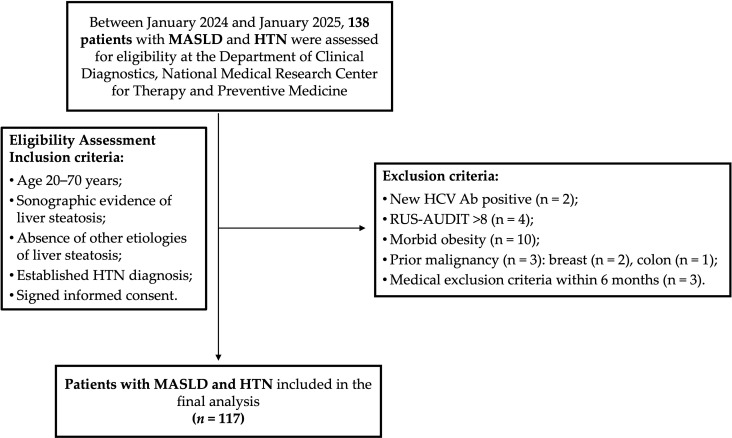
Participant flow chart. HCV Ab, antibodies to hepatitis C virus; HTN, hypertension; MASLD, metabolic dysfunction-associated steatotic liver disease; RUS-AUDIT, the Russian Alcohol Use Disorders Identification Test.

During the screening phase, 21 patients were excluded for the following reasons: 2 tested positive for hepatitis C virus antibodies (HCV Ab); 4 had a RUS-AUDIT score ≥ 8; 10 had morbid obesity; 3 reported a prior history of malignancy during clinical interviews (2 with previous breast cancer and 1 with colon cancer); and 3 had medical conditions meeting exclusion criteria that occurred approximately 2, 2.5, and 3 months before enrollment (Figure 1).

Therefore, 117 patients were included in the final analysis. Their demographic and clinical characteristics are presented in Table 1. The median age was 57 years (IQR, 49–64 years). Of the cohort, 85 patients (72.6%) were classified as obese, and 30 (25.6%) as overweight.

The distribution of hypertension (HTN) grades was grade 1 HTN, n = 40 (34.2%); grade 2 HTN, n = 33 (28.2%); and grade 3 HTN, n = 44 (37.6%). No statistically significant differences in gender or age were observed among patients with different HTN grades. Twelve patients (10.3%) had type 2 diabetes (T2D), and twenty-two (18.8%) had impaired fasting glycemia (IFG). Gender and age did not significantly differ between patients with and without T2D or IFG. Key findings from laboratory tests performed on patients with MASLD and HTN are summarized in Table 2.

The median Fatty Liver Index (FLI) was 84 [IQR, 68–94]. Of the patients, 99 (84.6%) had an FLI ≥ 60, and 16 (13.7%) had an FLI ≥ 30. The median liver stiffness value, as determined by pSWE, was 5.4 kPa [IQR, 4.1–6.3 kPa]. Elevated liver stiffness values (>7 kPa), suggestive of advanced liver fibrosis, were observed in 11 patients (9.4%; 8 males and 3 females; *p* = 0.031). Antihypertensive therapy was prescribed to 78 patients (66.7%; 34 males and 44 females; *p* = 0.792) (Table 3).

**Table 3:** The characteristics of the antihypertensive therapy received by patients with MASLD and HTN.

Antihypertensive Drug Class	Number of Patients with MASLD and HTN, n (%)
Calcium channel blockers	3.0 (2.6)
Beta blockers	9.0 (7.7)
Angiotensin receptor blockers	11.0 (9.4)
ACE inhibitors	8.0 (6.8)
Combination antihypertensive therapy	48 (41)

Note: abbreviations: ACE, angiotensin-converting enzyme; HTN, hypertension; MASLD, metabolic dysfunction-associated steatotic liver disease.

Among patients receiving combination antihypertensive therapy, 25 (52.1%) received a diuretic and a renin-angiotensin-aldosterone system (RAAS) blocker, 11 (22.9%) a calcium channel blocker and a RAAS blocker, 7 (14.6%) a beta blocker and a calcium channel blocker, and 5 (10.4%) a beta blocker and a RAAS blocker.

Based on muscle strength assessments, 34 patients (29.1%) were classified as having dynapenia, defined as either decreased grip strength (GS) (21, 17.9%; 18 females and 3 males; *p* = 0.003) or a FTSST completion time exceeding 15 s (13, 11.1%; 10 females and 3 males; *p* = 0.115). Of these 34 patients with MASLD and HTN, 2 (1.7%, both female) met both criteria for decreased muscle strength, exhibiting both decreased GS and a prolonged FTSST duration.

Based on established ALM/W index thresholds, 25 patients (21.4%; 11 females and 14 males; *p* = 0.126) in this cohort had decreased SMM, while 19 (16.2%; 15 females and 4 males; *p* = 0.037) met the criteria for sarcopenia, since they exhibited a simultaneous decline in muscle strength and SMM.

Osteopenia was present in 46 patients (39.3%; 36 females and 10 males; *p* = 0.001), and osteoporosis was diagnosed in only 3 patients (2.6%; 2 females and 1 male; *p* = 0.791) in this study.

When comparing subgroups of patients with MASLD and HTN, stratified by the presence or absence of the aforementioned musculoskeletal disorders and further categorized by HTN grade, T2D, or IFG status, we found that IFG was more prevalent among patients with dynapenia (defined by decreased GS: 38.1% in the dynapenia subgroup vs. 15.7% in the subgroup with preserved muscle strength; *p* = 0.015), sarcopenia (36.8% in the sarcopenia subgroup vs. 15.6% in those without sarcopenia; *p* = 0.022), and osteoporosis (present in 2 of 3 patients with osteoporosis [66.7%] vs. 16.7% in patients without osteoporosis; *p* = 0.027). The prevalence of T2D or HTN of any grade did not significantly differ between subgroups of MASLD patients with or without decreased muscle strength, SMM, or BMD. However, patients with MASLD and HTN diagnosed with decreased GS, sarcopenia, or osteopenia were significantly older than those with preserved muscle strength, SMM, and BMD (*p* = 0.013, *p* = 0.045, and *p* < 0.001, respectively).

### 3.2. Assessment of Serum Osteopontin and Procollagen Type 1 N-Terminal Propeptide Concentrations in Patients with Metabolic Dysfunction-Associated Steatotic Liver Disease and Hypertension

The median serum OPN concentration was 2.3 ng/mL (IQR, 1.44–3.8 ng/mL), and the median P1NP concentration was 44.1 pg/mL (IQR, 33.8–58.3 pg/mL). Serum OPN and P1NP levels did not significantly differ between male and female patients with MASLD and HTN (*p* = 0.160 and *p* = 0.184, respectively). Serum OPN and P1NP levels also did not significantly differ between subgroups of MASLD patients with and without T2D (*p* = 0.175 and *p* = 0.158, respectively) or IFG (*p* = 0.841 and *p* = 0.175, respectively). Furthermore, HTN grade was not significantly associated with serum OPN or P1NP concentrations in this cohort (ρ = 0.922 and *p* = 0.331, respectively). Serum OPN and P1NP concentrations did not significantly differ among subgroups of patients with dynapenia, sarcopenia, osteopenia, or osteoporosis. These differences remained non-significant after stratifying patients by gender.

#### 3.2.1. Correlation Analysis

Correlation analyses revealed statistically significant negative correlations between serum OPN concentrations and the following parameters: waist circumference (WC) (*ρ* = −0.334, *p* < 0.001), epicardial fat thickness (*ρ* = −0.249, *p* = 0.01), alanine aminotransferase (ALT) activity (*ρ* = −0.254, *p* = 0.006), aspartate aminotransferase (AST) activity (*ρ* = −0.248, *p* = 0.008), gamma-glutamyl transferase (GGT) activity (*ρ* = −0.217, *p* = 0.021), and liver stiffness (*ρ* = −0.209, *p* = 0.031). No statistically significant correlations were found between serum P1NP concentrations and any of the anthropometric, laboratory, or instrumental parameters evaluated in this study.

#### 3.2.2. Subgroup Analysis Depending on the OPN and P1NP Serum Concentrations

Due to the absence of established reference concentrations for serum OPN and P1NP, and to facilitate comparison between subgroups of patients with MASLD and HTN based on their serum OPN and P1NP concentrations, the mode serum concentrations of these osteokines—specifically, 2.89 ng/mL and 47.5 pg/mL, respectively—were used as threshold values (see “Statistical Analysis” section for more details). Forty-five patients (38.5%) with MASLD and HTN had serum OPN concentrations ≥ 2.89 ng/mL, whereas 47 (40.2%) had serum P1NP concentrations ≥ 47.5 pg/mL. Table 4 summarizes the characteristics of patient subgroups with MASLD and HTN, categorized by their serum OPN and P1NP concentrations.

**Figure 2:**
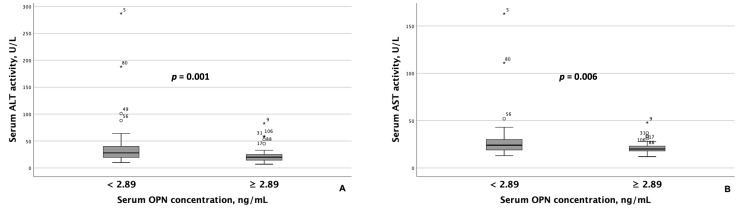
Patients with MASLD and HTN with serum OPN concentrations ≥ 2.89 ng/mL exhibited significantly lower serum ALT (**A**) and AST (**B**) activity compared to those with serum OPN concentrations < 2.89 ng/mL. Note: the central line within each box represents the median, and the box height represents the interquartile range. The whiskers depict the minimum and maximum values, and outliers are indicated by circles and asterisks. Comparisons were performed using the Mann–Whitney *U* test. Abbreviations: ALT, alanine transaminase; AST, aspartate aminotransferase; OPN, osteopontin.

**Figure 3:**
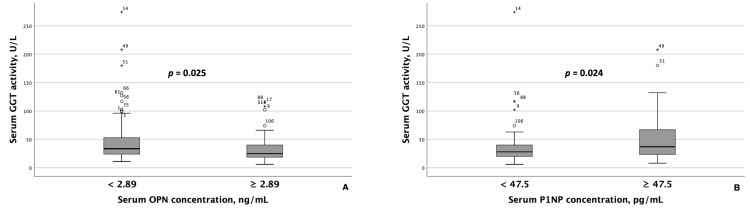
Serum GGT activity differed significantly based on serum OPN (**A**) and P1NP (**B**) concentrations in patients with MASLD and HTN. Note: the central line within each box represents the median, and the box height represents the interquartile range. The whiskers depict the minimum and maximum values, and outliers are indicated by circles and asterisks. Comparisons were performed using the Mann–Whitney *U* test. Abbreviations: GGT, gamma-glutamyl transferase; OPN, osteopontin; P1NP, procollagen type 1 N-terminal propeptide.

Patients with MASLD and HTN exhibiting serum OPN concentrations < 2.89 ng/mL had significantly higher AST and ALT activity than those with concentrations ≥ 2.89 ng/mL (Figure 2). Similarly, GGT activity was significantly higher among patients with lower serum OPN concentrations (Figure 3A). Although ALT and AST activity did not significantly differ between patients with serum P1NP concentrations < 47.5 pg/mL and ≥47.5 pg/mL, serum GGT activity was significantly higher among patients with MASLD and HTN and serum P1NP concentrations ≥ 47.5 pg/mL (Figure 3B).

**Figure 4:**
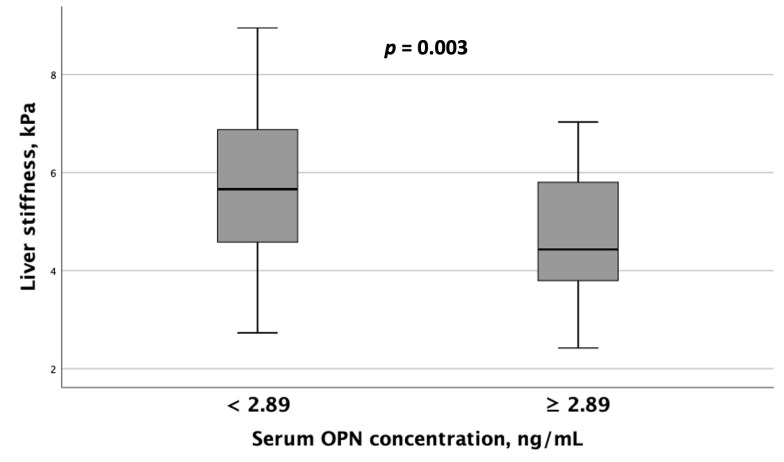
Patients with MASLD and HTN with serum OPN levels ≥ 2.89 ng/mL exhibited significantly lower liver stiffness values compared to those with serum OPN concentrations < 2.89 ng/mL. Note: the central line within each box represents the median, and the box height represents the interquartile range. The whiskers depict the minimum and maximum values, and outliers are indicated by circles and asterisks. Comparisons were performed using the Mann–Whitney *U* test. Abbreviations: OPN, osteopontin.

**Figure 5:**
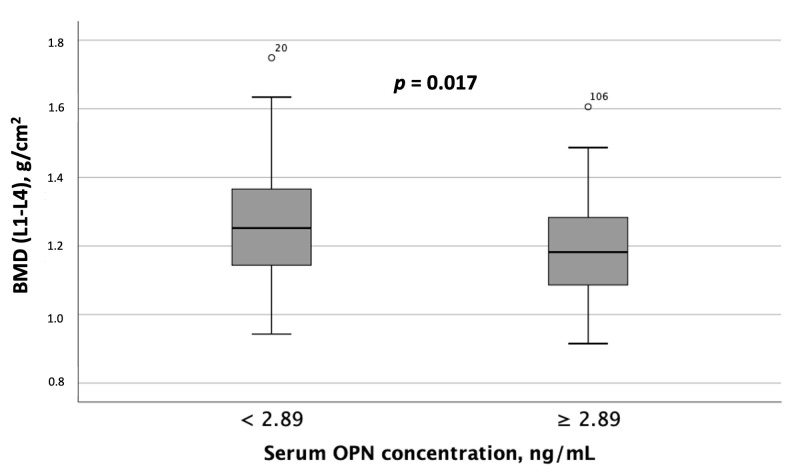
Spinal BMD (L1-L4) was significantly lower in patients with MASLD and HTN with serum OPN concentrations ≥ 2.89 ng/mL compared to those with serum OPN concentrations < 2.89 ng/mL. Note: the central line within each box represents the median, and the box height represents the interquartile range. The whiskers depict the minimum and maximum values, and outliers are indicated by circles. Comparisons were performed using the Mann–Whitney *U* test. Abbreviations: OPN, osteopontin.

When comparing instrumental test results, including liver elastography, SMM, and BMD measurements, between MASLD patient groups with differing serum OPN and P1NP concentrations, the following results were obtained. Patients with serum OPN concentrations ≥ 2.89 ng/mL exhibited significantly lower liver stiffness values (Figure 4). Furthermore, spinal BMD was substantially lower in this MASLD and HTN patient subgroup than in the subgroup with serum OPN concentrations < 2.89 ng/mL (Figure 5).

**Figure 6:**
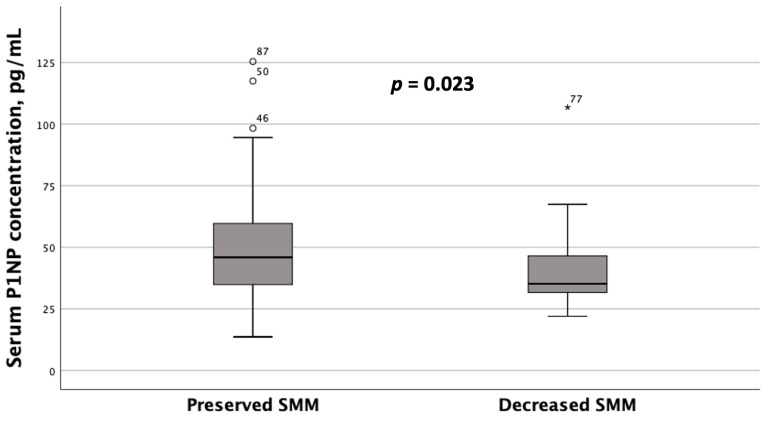
Serum P1NP concentrations in patients with MASLD and HTN with decreased and preserved skeletal muscle mass (SMM) (based on the DEXA results, namely appendicular skeletal muscle mass (ALM) to weight (W) index values). Note: the line through the middle of each box represents the median. The height of each box represents the interquartile range. The whiskers show the minimum and maximum values. Outliers are depicted as circles and asterisks. The comparison was performed using the Mann–Whitney *U* test.

Unlike OPN, subgroups with serum P1NP concentrations < 47.5 pg/mL and ≥47.5 pg/mL did not exhibit statistically significant differences in liver stiffness values or BMD at the spine or hip. However, patients with MASLD and HTN diagnosed with reduced SMM based on ALM/W index values had significantly lower serum P1NP concentrations compared to those with preserved SMM, as determined by DEXA (Figure 6). Serum OPN concentrations also showed a trend toward being lower in patients with decreased ALM/W index values, although this difference did not reach statistical significance (*p* = 0.060).

## 4. Discussion

Mounting evidence supports the association between MASLD and BMD, which appears independent of patient age or gender. For example, Z. Shen et al. demonstrated a multivariable-adjusted hazard ratio of 2.24 (95% CI: 1.18, 2.81) for incidents of low BMD in patients with MASLD compared to those without. These authors also noted that obesity and female gender may be considered risk factors for decreased BMD [42]. Moreover, a recent systematic review and meta-analysis indicated a significantly higher prevalence and risk of osteoporosis or osteoporotic fractures in both male and female patients with MASLD compared to those without the liver disease [9]. Lastly, the meta-analysis by A. Mantovani et al. indicated a significant association between MASLD and decreased whole-body BMD Z-scores in children and adolescents [43].

Given the established association between MASLD and decreased BMD, and considering both the importance of addressing osteopenia and osteoporosis in personalized lifestyle modification recommendations for patients with MASLD (particularly regarding physical activity) and the limitations of DEXA in diagnosing decreased BMD, we conducted a study to investigate serum concentrations of OPN and P1NP in patients with MASLD and HTN, examining their relationship with metabolic health indicators, biochemical and instrumental markers of liver disease severity, SMM, and BMD at the spine and hip in this patient population.

In summary, the findings of this study suggest an inverse relationship between serum OPN concentrations and surrogate laboratory markers of liver disease activity, including AST, ALT, and GGT, as well as with liver stiffness values in patients with MASLD and HTN. Y. Yilmaz et al. reported similar results, demonstrating a correlation between serum OPN concentrations and serum aminotransferase levels (aspartate aminotransferase: *β* = 0.295, *p* < 0.01; alanine aminotransferase: *β* = 0.285, *p* < 0.01) in patients with MASLD. The authors also proposed that serum OPN levels may serve as significant independent predictors of portal inflammation in this patient population [44]. The positive correlation between serum OPN concentrations and serum transaminase activity (markers of hepatocyte injury), as reported by Y. Yilmaz et al., may be attributed to the known proinflammatory effects of OPN. OPN, for example, acts as a chemoattractant for macrophages and regulates their migration, survival, phagocytosis, and pro-inflammatory cytokine production [45,46,47]. Conversely, in this study, we observed statistically significant inverse correlations between serum OPN concentrations and AST and ALT activity in the blood serum of patients with MASLD and HTN (*ρ* = −0.248, *p* = 0.008, and *ρ* = −0.254, *p* = 0.006, respectively). Furthermore, serum GGT activity was also inversely associated with serum OPN concentrations (*ρ* = −0.217, *p* = 0.021) in the MASLD and HTN patients included in this study. One potential explanation for the discrepancies between the Yilmaz et al. study [44] and the present study is that the patients in our study were older (median age 57 (49–64) years vs. 44 ± 10 years in the Yilmaz et al. study [44]), and approximately 42% exhibited decreased bone mineral density, indicative of osteopenia or osteoporosis. This age difference and the higher prevalence of decreased BMD may have influenced the relationship between serum AST and ALT activity and serum OPN concentrations in our study, given that OPN has been shown to be elevated in the blood serum of patients with osteopenia and osteoporosis. Consistent with this, serum OPN concentrations were substantially higher in patients with MASLD and HTN with decreased BMD at the spine in our study. Another potential explanation is that, in addition to bone cells (primarily osteoclasts), OPN is also expressed by macrophages, endothelial cells, smooth muscle cells, and epithelial cells [48]. Consequently, the presence of comorbidities, such as HTN, in the MASLD patients in our study could have influenced serum OPN concentrations and their associations with other laboratory parameters. Moreover, both our study and the Yilmaz et al. study [44] employed a cross-sectional design, which limits the ability to establish causal relationships between serum OPN concentrations and the presence or activity of MASLD. To the best of our knowledge, no longitudinal studies have examined serum OPN concentrations throughout the natural history of MASLD, nor have any assessed serum OPN concentrations in patients with MASLD across multiple consecutive visits during follow-up, which would be needed to elucidate the role of OPN in the development and progression of this liver disease.

In this study, we observed an inverse association between liver stiffness, a non-invasive measure of liver fibrosis progression, and serum OPN concentrations in patients with MASLD and HTN. In contrast, Yilmaz et al. [44] found no significant correlations between liver fibrosis stage (defined by histological assessment) and serum OPN levels. This discrepancy may be attributable to the fact that Yilmaz et al. [44] compared serum OPN levels across distinct fibrosis stages, with a limited number of patients in each stage, which likely hindered the detection of statistically significant differences.

Regarding P1NP, patients with MASLD and HTN with higher serum P1NP concentrations (≥47.5 pg/mL) exhibited higher GGT activity compared to those with lower concentrations, suggesting an association between this bone turnover marker and the presence of liver steatosis. These findings align with those of Deng et al., who demonstrated an independent correlation between serum P1NP concentrations and the presence of MASLD in a logistic regression analysis [49]. However, Fang et al. found that serum P1NP concentrations did not differentiate between the clinical forms of MASLD, namely steatosis and steatohepatitis [50]. Although serum P1NP concentrations did not significantly differ between MASLD patients with normal and elevated liver stiffness values, they were significantly higher in patients with MASLD and HTN who had elevated GGT activity, a surrogate marker for MASLD [51,52].

Unlike OPN, no significant differences were observed in other liver disease parameters or BMD at either the spine or hip between patients with serum P1NP concentrations ≥ 47.5 pg/mL and those with concentrations < 47.5 pg/mL. However, we found that patients with MASLD and HTN diagnosed with decreased SMM based on ALM/W index values had lower serum P1NP concentrations than those with preserved SMM, as determined by DEXA. Multiple studies have established a relationship between serum P1NP concentration and SMM in various patient populations. For example, in a study by Farup et al. involving 104 subjects undergoing bariatric surgery, serum P1NP concentrations were measured before and 6 to 12 months after the procedure. The authors observed an increase in serum P1NP concentration following bariatric surgery and concluded that this increase in the bone turnover marker may be associated with declining bone health and a predisposition to osteoporosis [53]. However, it is well documented that bariatric surgery also leads to rapid weight loss, including a reduction in skeletal muscle mass alongside body fat [54]. Therefore, one can infer that elevated serum P1NP levels in patients who have undergone bariatric surgery may reflect not only an increased risk of osteoporosis but also a decrease in SMM due to rapid weight loss resulting from caloric restriction [55]. Furthermore, Haeri et al. demonstrated an association between serum OPN concentrations and a frail phenotype (assessed using the Fried frailty index), a condition known to increase the risk of disability and adverse health outcomes, in 178 older women with osteoporosis [56].

Overall, the results of our study suggest that OPN and P1NP are not only involved in bone metabolism but also appear to play an important role in the development and natural course of MASLD. While we did not find a significant association between bone turnover markers and the stage of hypertension—possibly due to the limited sample size—we did identify associations between serum OPN concentrations and other components of the metabolic syndrome, such as waist circumference and epicardial fat thickness. This suggests that: (1) bone turnover markers may be involved in the development of metabolic disturbances observed in MASLD and HTN, and (2) metabolic dysregulation and the presence of MASLD and/or HTN may negatively impact BMD.

Currently, DEXA is the primary diagnostic tool for detecting decreased BMD. However, it has several limitations: it is not portable, has a weight limit of 130 kg (particularly relevant given that approximately 85% of MASLD patients are obese), and exposes both the patient and operator to ionizing radiation, albeit at very low doses [29].

Given the critical role of physical activity in managing MASLD and HTN, the known limitations of DEXA, and the potential adverse effects of certain exercises on bone health in patients at increased risk of osteopenia and osteoporosis, timely identification of decreased BMD in MASLD patients with or without HTN is essential. There is also a pressing need for non-invasive biomarkers to assess BMD in this population. Such markers could improve patient adherence to lifestyle modification recommendations and mitigate the risk of complications related to decreased BMD. Importantly, both OPN and P1NP are produced by various cell types across different tissues and organs. Therefore, their serum concentrations in MASLD patients may be influenced by coexisting diseases or conditions, as demonstrated in this study. Further research is needed in comorbid patient populations to validate the use of these bone-derived metabolites as surrogate markers of decreased BMD or impaired bone microarchitecture in clinical practice.

## 5. Limitations of the Study

This study has several limitations. First, the sample size is relatively small. However, the participants comprised a representative cohort of patients with MASLD and HTN who underwent a comprehensive clinical evaluation. Consequently, the findings can be cautiously extrapolated to the broader population of patients with MASLD, including those with coexisting HTN. Another limitation is the absence of a control group. However, the study was specifically designed to characterize serum OPN and P1NP concentrations in patients with MASLD and HTN, a prominent cardiometabolic comorbidity, and to analyze their associations with biochemical markers of liver disease severity, liver stiffness, body composition parameters, and BMD at the spine and hip. The study was not intended to compare these markers between MASLD patients and healthy individuals. Nevertheless, future comparative studies incorporating control groups would provide additional insights into the alterations in bone turnover markers in MASLD. To compare metabolic health indicators, non-invasive measures of liver disease severity, as well as muscle strength, SMM, and BMD between patients with “lower” and “higher” serum concentrations of OPN and P1NP, we used ad hoc cutoffs for OPN and P1NP based on visual inspection of their distribution rather than standardized clinical thresholds. Although this approach allowed us to perform exploratory subgroup analyses, the identified cutoffs were not derived from formal statistical modeling or clinical guidelines and should therefore be interpreted with caution. Future studies with larger cohorts are needed to validate clinically meaningful reference ranges for these biomarkers, ideally using standardized approaches such as percentile-based stratification, receiver operating characteristic (ROC) analysis, or outcome-based modeling. Finally, liver biopsy, the gold standard for diagnosing MASLD and assessing disease activity and fibrosis stage, was not performed. Instead, non-invasive methods recommended by the European Association for the Study of the Liver (EASL) were employed, i.e., standardized liver ultrasound and elastography [57].

## 6. Conclusions

In summary, the serum concentrations of OPN and P1NP in this study appear to be associated with various liver disease parameters, including ALT, AST, GGT activity, and liver stiffness, in patients with MASLD and HTN, which aligns with prior research [58,59]. Furthermore, the findings suggest an association between serum P1NP levels and SMM, as well as between serum OPN levels and spinal BMD, thereby indicating a link between liver function, muscle mass, and bone health. Moreover, OPN appears to be strongly associated with overall metabolic health indicators (such as WC and EFT), whereas P1NP is more closely linked to muscle mass.

## Figures and Tables

**Table 1:** Demographic and anthropometric parameters of patients with MASLD and HTN.

Parameter	Patients with MASLD and HTN (n = 117)
Female gender, n (%)	67 (57.3)
Age, years	57 (49–64)
BMI, kg/m^2^	32.9 (29.6–36.2)
Normal weight (BMI 18.5–24.9 kg/m^2^), n (%)	2 (1.7)
Overweight (BMI 25.0–29.9 kg/m^2^), n (%)	30 (25.6)
Obesity (BMI ≥ 30 kg/m^2^), n (%)	85 (72.6)
Waist circumference (all subjects), cm	108 (102–115)
Waist circumference (male), cm	111.5 (106–117)
Normal Range	≤94 cm
Patients Outside the Normal Range, n (%)	50 (100)
Waist circumference (female), cm	105 (98–111)
Normal Range	≤80 cm
Patients Outside the Normal Range, n (%)	66 (98.5)
Hip circumference, cm	113 (108–118.75)

Note: numerical data are presented as median [interquartile range]; categorical variables are presented as n (%). Abbreviations: BMI, body mass index; HTN, hypertension; MASLD, metabolic dysfunction-associated steatotic liver disease.

**Table 2:** Results of laboratory tests in patients with MASLD and HTN.

Parameter	Patients with MASLD and HTN (n = 117)	Normal Range	Patients Outside the Normal Range, n (%)
Erythrocytes, 10^12^/L	4.80 (4.51–5.00)		
Erythrocytes, 10^12^/L (male)	4.98 (4.76–5.28)	4.0–5.9	0 (0)
Erythrocytes, 10^12^/L (female)	4.6 (4.3–4.8)	3.8–5.2	0 (0)
Hemoglobin, g/L	146 (133–152)		
Hemoglobin, g/L (male)	152 (147–156.5)	140–175	0 (0)
Hemoglobin, g/L (female)	137 (129–145.5)	123–153	0 (0)
ESR, mm/h	9 (4–14)	<20	0 (0)
Platelets, 10^9^/L	253.0 (211.8–290.0)	150–450	0 (0)
Leukocytes, 10^9^/L	6.4 (5.3–7.5)	5–10	0 (0)
ALT, IU/L	23.0 (16.0–37.3)	0–34	34 (29.1)
AST, IU/L	22.0 (18.0–26.3)	0–34	10 (8.5)
GGT, IU/L	31.0 (21.0–45.5)	0–38	42 (35.9)
Albumin, g/dL	4.5 (4.3–4.6)	3.5–5.2	0 (0)
Total bilirubin, μmol/L	11.7 (9.0–14.5)	3.4-20.5	6 (5.1)
Uric acid, μmol/L	357 (327.3–416.5)	154.7–356.9	57 (48.7)
CRP, mg/L	2.2 (1.4–5.0)	0–5	29 (24.8)
Total cholesterol, mmol/L	5.8 (4.9–6.2)	0–5	82 (70.1)
Triglycerides, mmol/L	1.43 (1.10–2.10)	<1.7	46 (39.3)
Creatinine, μmol/L	75.7 (67.6–90.0)	51–98	10 (8.5)
Glucose, mmol/L	5.7 (5.4–6.0)	3.9-5.5	72 (61.5)
Calcium, mmol/L	2.4 (2.3–2.5)	2.1–2.55	4 (3.5)
Phosphorus, mmol/L	1.1 (0.9–1.2)	0.81-1.45	0 (0)
HOMA-IR > 2.7, n (%)	61.0 (52.1)	0.5–1.4	-
pSWE, kPa	5.4 (4.1–6.3)	<7	11 (9.4)

Note: numerical data are presented as median [interquartile range]; categorical variables are presented as n (%). Abbreviations: ALT, alanine aminotransferase; AST, aspartate aminotransferase; CRP, C-reactive protein; ESR, erythrocyte sedimentation rate; GGT, gamma-glutamyl transferase; HOMA-IR, Homeostasis Model Assessment of Insulin Resistance; HTN, hypertension; MASLD, metabolic dysfunction-associated steatotic liver disease; pSWE, point shear-wave elastography.

**Table 4:** The characteristics of patient subgroups with MASLD and HTN, categorized by their serum OPN and P1NP concentrations.

Parameter	Serum OPN Concentration in Patients with MASLD and HTN, ng/mL			Serum P1NP Concentration in Patients with MASLD and HTN, pg/mL		
	<2.89 (n = 72)	≥2.89 (n = 45)	*p*	<47.5 (n = 70)	≥47.5 (n = 47)	*p*
Age, years	55.5 (47.3–62.8)	58.0 (51.0–64.5)	n/s	57.0 (49.0–65.0)	56.0 (48.0–63.0)	n/s
Gender: female, n (%)	38.0 (52.8)	29.0 (64.4)	n/s	38.0 (54.3)	29.0 (61.7)	n/s
BMI, kg/m^2^	33.2 (30.6–36.1)	32.1 (28.9–36.9)	n/s	32.9 (30.1–36.1)	32.8 (29.5–36.8)	n/s
HTN, n (%):			n/s			n/s
Grade 1	24.0 (33.3)	15.0 (33.3)		23.0 (32.9)	16.0 (34.0)	
Grade 2	21.0 (29.2)	13.0 (28.9)		22.0 (31.4)	12.0 (25.5)	
Grade 3	27.0 (37.5)	17.0 (37.8)		25.0 (35.7)	19.0 (40.5)	
Antihyperten-sive therapy, n (%)	47.0 (65.3)	31.0 (68.8)	n/s	43.0 (61.4)	35 (74.5)	n/s
T2D, n (%)	8.0 (11.1)	4.0 (8.8)	n/s	9.0 (12.9)	3.0 (6.4)	n/s
IFG, n (%)	13.0 (18.1)	9.0 (20)	n/s	12.0 (17.1)	10.0 (21.3)	n/s

Note: quantitative data are presented as median [interquartile range]; categorical variables are presented as n (%). Quantitative data were compared using the Mann–Whitney *U* test, and categorical data were compared using the chi-squared test. Abbreviations: n/s, not significant; BMI, body mass index; HTN, hypertension; IFG, impaired fasting glycemia; MASLD, metabolic dysfunction-associated steatotic liver disease; T2D, type 2 diabetes mellitus.

## Data Availability

The data presented in this study are available upon reasonable request from the corresponding authors. The data are not publicly available due to privacy restrictions.
